# Arginase Pathway in Acute Retina and Brain Injury: Therapeutic Opportunities and Unexplored Avenues

**DOI:** 10.3389/fphar.2020.00277

**Published:** 2020-03-17

**Authors:** Abdelrahman Y. Fouda, Wael Eldahshan, S. Priya Narayanan, R. William Caldwell, Ruth B. Caldwell

**Affiliations:** ^1^Vascular Biology Center, Medical College of Georgia, Augusta University, Augusta, GA, United States; ^2^Culver Vision Discovery Institute, Medical College of Georgia, Augusta University, Augusta, GA, United States; ^3^Charlie Norwood VA Medical Center, Augusta, GA, United States; ^4^Clinical Pharmacy Department, Faculty of Pharmacy, Cairo University, Giza, Egypt; ^5^Department of Pharmacology and Toxicology, Medical College of Georgia, Augusta University, Augusta, GA, United States; ^6^Department of Clinical and Administrative Pharmacy, University of Georgia, Athens, GA, United States; ^7^Department of Cellular Biology and Anatomy, Medical College of Georgia, Augusta University, Augusta, GA, United States

**Keywords:** arginase, polyamines, retina, brain, ischemic injury, traumatic injury

## Abstract

Ischemic retinopathies represent a major cause of visual impairment and blindness. They include diabetic retinopathy (DR), acute glaucoma, retinopathy of prematurity (ROP), and central (or branch) retinal artery occlusion (CRAO). These conditions share in common a period of ischemia or reduced blood supply to the retinal tissue that eventually leads to neuronal degeneration. Similarly, acute brain injury from ischemia or trauma leads to neurodegeneration and can have devastating consequences in patients with stroke or traumatic brain injury (TBI). In all of these conditions, current treatment strategies are limited by their lack of effectiveness, adverse effects or short time window for administration. Therefore, there is a great need to identify new therapies for acute central nervous system (CNS) injury. In this brief review article, we focus on the pathway of the arginase enzyme as a novel therapeutic target for acute CNS injury. We review the recent work on the role of arginase enzyme and its downstream components in neuroprotection in both retina and brain acute injury models. Delineating the similarities and differences between the role of arginase in the retina and brain neurodegeneration will allow for better understanding of the role of arginase in CNS disorders. This will also facilitate repurposing the arginase pathway as a new therapeutic target in both retina and brain diseases.

## Introduction

The brain and the retina are primary components of the central nervous system (CNS). In fact, the retina is developed from the brain tissue during embryogenesis. Therefore, it is considered an extension of the brain (London et al., 2013). Both the retina and the brain are neurovascular organs with supporting glia cells. It has been recently appreciated that disease mechanisms in the brain can be extended to the retina and vice versa (London et al., 2013; Jindal, 2015). With the retina and the brain being windows to each other, reviewing the literature on both organs greatly improves our understanding of the mechanisms of CNS injury and neurodegeneration.

Arginase is an important enzyme that has been conserved across species, including plants, bacteria, invertebrates, and vertebrates (Dzik, 2014). It has long been known for its central role in ammonia detoxification in the urea cycle of the liver (Wu and Morris, 1998). Furthermore, arginase has been shown to play an important role in different pathological conditions of the kidney, cardiovascular system, immune system, neurovascular system and certain types of cancers (Caldwell et al., 2018).

Several studies have linked arginase to CNS disorders such as Alzheimer’s, multiple sclerosis, diabetic retinopathy (DR), retinopathy of prematurity (ROP), among others. In this brief review, we focus on the role of arginase in acute retina and brain injury. In addition, we briefly discuss the recent literature on the role of arginase in Alzheimer’s disease (AD) and tauopathies, which is an area of increasing interest in recent years. The reader is referred to our recent reviews for discussion of the role of arginase in other chronic CNS disorders (Caldwell et al., 2015, 2018).

## The Arginase Pathway

Arginase is the enzyme that converts the amino acid, arginine, to ornithine and urea. Ornithine, the product of the arginase enzymatic reaction is further converted by ornithine decarboxylase (ODC) to form the polyamines, putrescine, spermine and spermidine and by the ornithine aminotransferase pathway to form proline (Caldwell et al., 2018). Ornithine can also be produced from glutamate via ornithine aminotransferase independent of arginase (Ginguay et al., 2017). There are two isoforms of arginase, arginase 1 (A1) and arginase 2 (A2) that are encoded by two different genes. Human A1 is a 322 amino acid protein while A2 has 354 amino acids (Dizikes et al., 1986; Gotoh et al., 1996). A1 and A2 amino acids sequences share more than 60% homology, however, they are identical in the enzyme catalytic site (Vockley et al., 1996). While the two isoforms mediate the same enzymatic reaction, they differ in their tissue and cell-specific expression as well as subcellular localization. A1 is cytosolic and it is highly expressed in liver tissue where it plays a major role in ammonia detoxification through the urea cycle. A2 is present in the mitochondria. It is highly expressed in the kidney. Both isoforms are expressed in other peripheral tissues and in the CNS, including the retina and the brain. The arginase isoforms and their downstream products polyamines and proline play diverse physiological roles and are induced or downregulated under pathological conditions (Morris, 2009; Caldwell et al., 2018). The polyamines can play important roles in cell proliferation, ion channel function, and neuroprotection. Proline is involved in collagen formation which can contribute to wound healing and/or fibrosis (Wu and Morris, 1998; Caldwell et al., 2018). Apart from their role in generation of polyamines and prolines, the arginases can compete with the nitric oxide synthase (NOS) enzyme for the common substrate arginine. Therefore, arginase can regulate the function of the three NOS isoforms, endothelial (eNOS), inducible (iNOS), and neuronal (nNOS).

## Role of the Arginase Pathway in Acute Retina Injury

For the sake of this review, we focus on two acute models of retinal injury, ischemia-reperfusion injury (IR) and optic nerve crush (ONC), a model for traumatic optic neuropathy (TON). Both models involve neurodegeneration in the form of loss of inner retinal neurons within one to 2 weeks of injury. Our work using both the IR injury and ONC models showed retinal neuroprotection in A2 knockout (KO) mice (Shosha et al., 2016; Xu et al., 2018). These studies show that A2 plays a central role in the pathophysiology of acute retinal injury. In the IR injury model, which leads to both neuronal and microvascular degeneration, A2 deletion reduced neurodegeneration and limited the formation of acellular capillaries. Furthermore, A2 deletion reduced oxidative stress and cell death by necroptosis. This protective effect of A2 deletion after IR injury was confirmed by the improved retina function as shown by electroretinographic recording in A2 KO mice (Shosha et al., 2016).

In the ONC model, A2 deletion reduced death of the retinal ganglion cells (RGCs) and prevented degeneration of their axons, while enhancing axonal sprouting. In addition to the decreased inflammatory response, A2 KO retinas showed increases in the neurotrophin, brain derived neurotrophic factor (BDNF) and its downstream signaling following ONC. In both the IR injury and ONC models, A2 deletion preserved retinal structure and reduced glial activation.

In contrast to A2, our studies with A1 knockout mice showed a worsened neurovascular outcome after IR injury with A1 deletion either globally or specifically in myeloid cells (Fouda et al., 2018). This worsened outcome was manifested in the form of retinal neurodegeneration and thinning as well as increased acellular capillaries. A1 deletion was also associated with increased retinal inflammatory response and cell death by necroptosis after IR. On the other hand, Intravitreal treatment with pegylated A1 (PEG-A1), a stable form of the enzyme, improved neuronal survival in WT mice after IR. Our *in vitro* studies showed an increased inflammatory response in A1KO macrophages which was ameliorated with PEG-A1 treatment. Intravitreal administration of A1KO macrophages worsened the retinal IR injury (Fouda et al., 2018). Collectively these data show a protective role of myeloid A1 against retinal IR injury. Interestingly, when A1 competes with inducible NOS (iNOS) and reduces L-arginine available to it, it dampens iNOS-induced inflammation and oxidative stress (Kleinert et al., 2003; Fouda et al., 2018). A recent report has indicated that A1 expression in macrophages is critical for suppression of iNOS expression (Suwanpradid et al., 2017). The role of A1 in the ONC model is yet to be elucidated.

Unlike A1, A2 is upregulated in pro-inflammatory macrophage and has been shown to promote the macrophage inflammatory response (Ming et al., 2012; Yang and Ming, 2014). However, the role of A2 in macrophages is still controversial, with some reports suggesting an anti-inflammatory role of A2 in macrophages (Johann et al., 2007; Lewis et al., 2011; Hardbower et al., 2016). Therefore, the role of A2 in macrophages is as yet unclear. It is possible that there is a reciprocal regulation between A1 and A2 in macrophages and other cell types that can explain the opposite roles of the two isoforms in retinal IR injury. This interaction between the two isoforms needs to be further explored.

### Polyamines in Acute Retinal Injury

Given that both arginase isoforms mediate the same enzymatic reaction, a central unanswered question about the differential roles of A1 and A2 in acute retinal injury is how each isoform affects the downstream polyamine metabolism in different cell types. The role of polyamines has been studied in ONC and retinal excitotoxicity models. In the ONC model, oral administration of the polyamine, spermidine, in drinking water protected against retinal ganglion cell (RGC) loss and retinal degeneration while enhancing optic nerve regeneration. The spermidine treatment decreased the ONC-induced apoptotic signaling, reduced iNOS expression and limited microglia accumulation (Noro et al., 2015b). Similarly, oral administration of spermidine has been shown to ameliorate neurodegeneration in a genetic mouse model of normal tension glaucoma that involves glutamate neurotoxicity (Noro et al., 2015a). Polyamine treatment is also protective against monosodium glutamate neurotoxicity in the newborn rat retinas (Gilad and Gilad, 1989). However, another study showed that intravitreal injection of the polyamines, putrescine or spermine exacerbated RGC loss in a model of N-methyl-D-aspartate (NMDA)-induced excitotoxic injury. This damage was attributed to binding of polyamines to the NMDA receptor (Pernet et al., 2007). Taken together, the role of polyamines in acute retinal injury seems to be dependent on the injury model and route of administration, and therefore needs further elucidation.

In addition, polyamine metabolism can be dysregulated under pathological conditions via induction of certain enzymes such as polyamine oxidase (PAO) leading to increases in toxic polyamine oxidative products. The role of PAO in retinopathy has been recently reviewed by Narayanan et al. (2019). Studies performed by Pichavaram et al. demonstrated the critical involvement of polyamine oxidation in a model of excitotoxicity-induced retinal neuronal damage (Pichavaram et al., 2018). In that study, expression of the PAO, spermine oxidase, was shown to be upregulated in response to NMDA treatment. Furthermore, loss of RGCs, degeneration of synaptic contacts, and loss of inner retinal neurons were evident in NMDA-treated retinas. Treatment with MDL 72527, a selective enzyme-activated irreversible inhibitor of PAO significantly reduced these effects and improved neuronal survival.

## Role of the Arginase Pathway in Acute Brain Injury

There have been few studies that directly addressed the role of arginase after acute brain injury that occurs with stroke or traumatic brain injury (TBI). Both arginase isoforms have been shown to be expressed in rat brain neurons at basal conditions. After photothrombotic stroke, A1 expression was increased in macrophages and astrocytes in the vicinity of the ischemic lesion while A2 expression was unchanged. Moreover, A1 expression co-localized with the neurotrophin, BDNF, in activated astrocytes. This was associated with an increase in overall arginase activity post-stroke (Quirie et al., 2013). A more recent study showed exclusive expression of A1 in infiltrating myeloid cells (macrophages) but not microglia in the spinal cord after contusion injury or experimental autoimmune encephalomyelitis (EAE) induction (Greenhalgh et al., 2016). Similarly, the same group showed that A1 is expressed by macrophages rather than microglia after permanent middle cerebral artery occlusion (Zarruk et al., 2018).

A1 has been used extensively in stroke studies as a marker for the M2-like anti-inflammatory macrophages with its expression in myeloid cells being correlated with better outcomes. After experimental stroke, the number of A1 positive macrophages correlated with neuroprotection and functional recovery (Hamzei Taj et al., 2016). However, the direct role of A1 in stroke pathophysiology has not been rigorously examined to our knowledge. On the other hand, Ahmad et al. (2016) tested the role of A2 in ischemic stroke using a permanent middle cerebral artery occlusion model. A2 KO mice showed increased brain infarction and worse neurological deficit at 7 days after stroke as compared to wild type (WT) but there was no significant difference in cerebral blood flow between the two groups post-ischemia. The authors further confirmed the protective role of A2 using the NMDA-induced acute excitotoxicity model. However, the study did not address the underlying mechanism of this protective effect of A2. A more recent study showed upregulation of both arginase isoforms in rat brain after stroke and improvement of stroke outcomes with the indirect arginase inhibitors, L-citrulline, L-ornithine, and L-norvaline (Barakat et al., 2018). However, while these amino acids can modulate arginase activity, they are not specific for arginase and can have other off-target effects.

Similar to stroke, A1 has been shown to be upregulated in macrophages but not microglia after TBI (Hsieh et al., 2013). Treatments that improved TBI outcomes also increased M2-like, A1 positive macrophages (Gao et al., 2017; Xu et al., 2017). Overexpression of A1, but not A2, in brain neurons protected against TBI (Madan et al., 2018). Another study showed improved recovery of cerebral blood flow after TBI in A2 knockout mice (Bitner et al., 2010). Taken together, the role of arginase in acute brain injury and the interplay between the two enzyme isoforms still require further investigation.

### The Polyamines and Brain Injury

In mice subjected to experimental stroke, polyamines were found to be decreased within the infarct area but increased systemically in plasma at 24 h (Saiki et al., 2009). Inhibition of ODC with DFMO (difluoromethylornithine) reduced stroke infarct volume in rats, suggesting a deleterious role of polyamines in stroke (Muszynski et al., 1993). However, DFMO also inhibits arginase due to feed-back inhibition associated with the accumulation of ornithine (Selamnia et al., 1998). In contrast, treatment with spermine but not putrescine or spermidine was neuroprotective in a neonatal rat model of hypoxia-ischemia. Interestingly, spermine treatment was associated with inhibition of arginase activity and increased NOS activity, suggesting involvement of arginase in the pathology (Clarkson et al., 2004). In TBI models, polyamine catabolism has been found to be increased in the brain with a six-fold increase in putrescine levels but not spermine and spermidine within the first 3 days after injury (Shohami et al., 1992; Henley et al., 1996; Doğan et al., 1999a; Zahedi et al., 2010). Treatment with DFMO improved the neurological outcome after TBI in rats but did not affect edema formation or blood-brain barrier breakdown (Shohami et al., 1992). Further study is needed to elucidate the specific mediator(s) of these DFMO effects.

Again, as with retinal injury, the role of polyamines in acute brain injury is further complicated by the induction of PAO enzyme activity, leading to the formation of the toxic products, acrolein, and 3-aminopropanal that have been shown to strongly correlate with stroke pathology (Ivanova et al., 1998; Saiki et al., 2009). Acrolein is a highly reactive aldehyde and a potent mediator of oxidative damage. DNA adduction, inflammation, membrane disruption, protein adduction, and endoplasmic reticulum stress are the major mechanisms of acrolein-induced toxicity (Moghe et al., 2015). Expression of spermine oxidase was found to be increased in a rat model of stroke (Fan et al., 2019), and this increase has been shown to be positively correlated with the severity of brain injury following excitotoxic damage (Cervetto et al., 2016; Pietropaoli et al., 2018). Earlier studies demonstrated that blockade of polyamine oxidation using the PAO inhibitor, MDL 72527, was neuroprotective in a rat stroke model (Doğan et al., 1999b). Similarly, MDL 72527 reduced traumatic injury volume and brain edema in a rat model of TBI (Doğan et al., 1999a). Collectively, these studies show a deleterious role of PAO in acute brain injury.

## Clinical Studies Examining the Arginase-Polyamine Pathway in Acute Retina and Brain Injury

To our knowledge, the role of arginase pathway in CNS injury has not been tested clinically and only a few studies have described changes in the pathway in patient samples. A1 mRNA was shown to be acutely upregulated in peripheral blood of ischemic stroke and mild TBI patients (Petrone et al., 2017; Yoo et al., 2019). Furthermore, increased serum A1 levels were found to be correlated with ischemic stroke severity and post-stroke immune suppression as measured by neutrophil-lymphocyte ratio (NLR) (Petrone et al., 2016). Similarly, plasma polyamine, PAO, and acrolein levels were shown to be upregulated acutely after ischemic stroke and this effect was correlated with infarct volume and clinical outcome (Els et al., 2001; Tomitori et al., 2005). Therefore, the use polyamine metabolites as biochemical markers for stroke has been proposed (Igarashi and Kashiwagi, 2011).

To our knowledge, levels of the two different arginase isoforms have not been studied in patients with any form of retinopathy. However, metabolomic profiling of vitreous samples from patients with proliferative DR revealed increases in arginase pathway metabolites, including arginine, ornithine, and proline with the later showing the most pronounced increase (Paris et al., 2015). Furthermore, L-arginine pathway is dysregulated in plasma samples from type 2 diabetic patients with PDR (Sumarriva et al., 2019; Zhu et al., 2019). In addition, another study of vitreous samples collected from patients with proliferative DR found increases in spermine and spermidine levels as compared to non-diabetic controls while putrescine was reduced (Nicoletti et al., 2003). Collectively, these studies show the strong involvement of the arginase pathway in the pathophysiology of retinopathy in humans.

## Arginase in Alzheimer’s Disease and Tauopathies

Alzheimer’s disease is a neurodegenerative disorder characterized by the accumulation of β-amyloid (Aβ) peptides, and hyper-phosphorylated and aggregated tau in the brain. Tau is a protein that promotes microtubule assembly and stabilization in axons (Hunt et al., 2015). Post-translational modifications of tau result in its dysfunction and a group of neurodegenerative diseases termed tauopathies (Lee et al., 2001; Hunt et al., 2015). Overexpression of A1 in the hippocampus of a mouse model of tauopathy resulted in a decrease in tau pathology and hippocampal atrophy. One mechanism is through inhibiting mTOR signaling pathway and stimulation of autophagy (Hunt et al., 2015). A second mechanism could be through the formation of polyamines which stabilize already destabilized microtubules in tau pathology (Hunt et al., 2015; Vemula et al., 2019). Surprisingly, A1 has been shown to accumulate at sites of Aβ deposition (Kan et al., 2015). In murine models of AD, inhibition of arginase using DFMO or L-norvaline reduced Aβ deposition (Kan et al., 2015; Polis et al., 2018). However, these compounds inhibit both isoforms of arginase making it difficult to study the effect of individual isoforms on AD pathology.

There is evidence from clinical studies that the urea cycle is involved in the pathophysiology of AD (Hansmannel et al., 2010). Of all the urea cycle enzymes, ornithine transcarboxylase (OTC), is differentially expressed in vascular endothelial cells of AD brains compared to controls indicating an active urea cycle (Bensemain et al., 2009). The increase in OTC expression could be a compensation for the decrease in glutamine synthase (GS) activity in the frontal pole of AD brains that results in ammonia build up (Smith et al., 1991). Additionally, an increase in arginase activity is reported in AD brains consistent with an increase in A2 expression and no significant change in A1 (Hansmannel et al., 2010; Liu et al., 2014). In the plasma, however, a decrease in arginase activity has been reported in patients with AD (Vural et al., 2009). The expression of plasma arginase isoforms was not measured in this study. These data indicate the overall involvement of arginase in AD pathology in humans. DFMO use did not slow cognitive decline in a single-case study of an AD patient (Alber et al., 2018). Short term L-arginine administration improved cognitive function in a small group of elderly (Ohtsuka and Nakaya, 2000). A Recent clinical trial in cognitively intact older adults showed enhanced memory performance with spermidine supplementation (Wirth et al., 2018). Despite this, the role of arginase and polyamines in AD has not been directly tested in clinical trials and there is no evidence so far that related therapies can stop the progression of AD in humans.

Taken together and based on clinical and pre-clinical data, we speculate that the build-up of A1 at sites of Aβ deposition and tau dysfunction could be a compensatory mechanism to halt disease progression. Accumulation of A2, however, could be a negative consequence that further exacerbates the disease pathology.

## Arginase Pathway as a Therapy for Acute CNS Injury and AD

**FIGURE 1:**
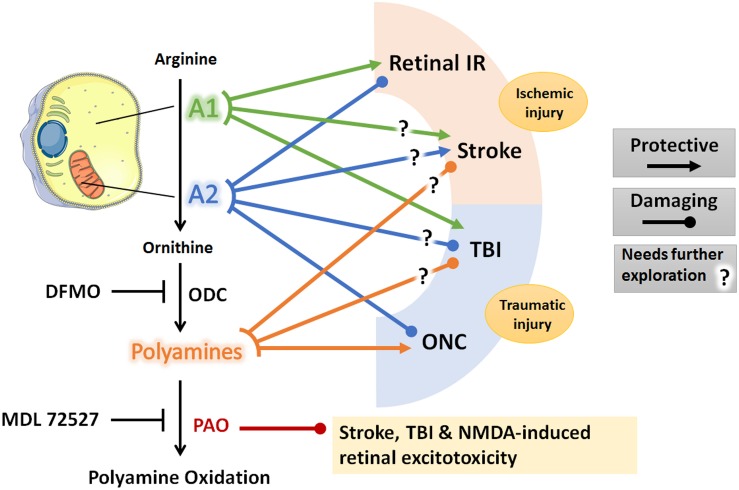
Schematic diagram of the arginase pathway components and their role in retina and brain ischemic and traumatic injuries. Lines denote a protective or damaging role of each component on the listed CNS injury models based on animal studies reviewed in the manuscript. Question marks denote conflicting experimental evidence. Diagram was created using Servier^®^ medical art https://smart.servier.com/. A1, arginase 1; A2, arginase 2; IR, ischemia-reperfusion; ONC, optic nerve crush; TBI, traumatic brain injury; ODC, ornithine decarboxylase; PAO, polyamine oxidase; DFMO, difluoromethylornithine; NMDA, N-methyl-D-aspartate.

Based on the above reviewed work, arginase pathway represents a potential therapeutic target in acute CNS injury. The role of arginase pathway components in acute brain and retinal injury is summarized in Figure 1. Specifically, use of A1 as a therapy for acute CNS ischemia and AD is an appealing approach since PEG-A1 is already being clinically tested in patients with advanced hepatocellular carcinoma and melanoma, and it appears to be safe and well-tolerated (Yau et al., 2013, 2015; De Santo et al., 2018). However, possible systemic adverse effects on endothelial cell function and immune responses should also be tested and considered. One study showed that experimental TBI induces endothelial dysfunction in the systemic microcirculation via A1 upregulation and eNOS uncoupling (Villalba et al., 2017). Similarly, another study showed that A1 release by neutrophils induced peripheral immunosuppression in a murine stroke model (Sippel et al., 2015). This was echoed by clinical data showing a correlation between increased serum A1 levels and post-stroke immune suppression (Petrone et al., 2016). Therefore, the timing and duration of the administration of arginase needs to be studied in detail in order to harness the protective effect of this drug in CNS disease with a high efficacy and safety profile. In addition to A1, manipulation of A2, polyamine levels, or PAO can also be targeted to limit acute CNS injury. However, there is no clinically tested drug that can specifically target A2 or PAO. DFMO, which inhibits ODC and therefore can reduce polyamine levels, has been tested in neuroblastoma patients and found to be protective (Sholler et al., 2018). As was noted above, DFMO lacks specificity and can also inhibit arginase activity. More research into the definitive role of polyamines in acute CNS injury is warranted before further pursuing this pathway as a therapeutic target.

## Author Contributions

AF drafted the main body of the mini-review. RWC, SN, and WE helped with drafting and editing. RBC revised and approved the final version.

## Conflict of Interest

AF, RBC, and RWC have a pending patent on the use of arginase 1 as a treatment for ischemic retinopathies.

The remaining authors declare that the research was conducted in the absence of any commercial or financial relationships that could be construed as a potential conflict of interest.
